# Supplementary material for: Aspiration Pneumonia After ERCP Under Anesthesiologist-Administered Sedation: Prevalence, Risk Factors and Clinical Outcomes of an Underestimated Adverse Event
Source: Medicina (Kaunas). 2025 Dec 6;61(12):2172. doi: 10.3390/medicina61122172 (PMC12735329; doi:10.3390/medicina61122172)
Supplement: Supplementary file 1 [file medicina-61-02172-s001.zip › medicina-3975872-supplementary.pdf]

Supplementary:.

Table S1. (Supplementary Material): Unadjusted analysis on the incidence of 30-day mortality (30d mortality).

p-value	30d mortality n (%)	Patients n (%)	
/	/	**1140 (100)**	**Included Patients**
			**Sex**
0.051	17 (3.5)	487 (42.7)	*Females*
	11 (1.7)	653 (57.3)	*Males*
			**Age group**
0.028	2 (0.7)	279 (24.5)	*<60 years*
	10 (3.2)	316 (27.7)	*60-69 years*
	5 (1.5)	334 (29.3)	*70-79 years*
	11 (5.2)	211 (18.5)	*>=80 years*
			**Indication to ERCP**
<0.001	25 (4.8)	572 (50.2)	*Malignant stricture*
	2 (0.7)	270 (23.7)	*Biliary Stones*
	0 (0)	170 (14.9)	*Benign stricture*
	1 (0.8)	128 (11.2)	*Other*
			**Comorbidity**
0.083	19 (3.2)	589 (51.7)	Yes
	9 (1.6)	551 (48.3)	No
			Diabetes
0.915	5 (2.3)	220 (19.6)	*Yes*
	23 (2.4)	920 (80.4)	*No*
			Cardiovascular
<0.001	15 (5.0)	299 (26.2)	*Yes*
	12 (1.4)	840 (73.8)	*No*
			Pulmonary
0.859	2 (2.0)	95 (8.3)	*Yes*
	26 (2.4)	1044 (91.7)	*No*
			**ASA-score**
0.003	2 (0.6)	318 (27.9)	1
	13 (2.4)	549 (48.2)	2
	11 (4.4)	252 (22.1)	3
	1 (5.0)	20 (1.8)	4
			**Cholangitis**
0.921	2 (2.3)	87 (7.6)	*Yes*
	26 (2.5)	1053 (92.4)	*No*
			**Previous sphyncterotomy**
0.501	10 (2.1)	477 (41.9)	*Yes*
	18 (2.7)	661 (58.1)	*No*
			**Cannulation**
0.005	22 (2.1)	1053 (92.4)	*Yes*
	6 (6.9)	87 (7.6)	*No*
			**Difficult cannulation**
0.063	11 (4.0)	278 (24.4)	*Yes*
	17 (2.0)	861 (75.6)	*No*
			**Double wire**
0.054	5 (5.4)	92 (8.1)	*Yes*
	23 (2.2)	1048 (91.9)	*No*

			**Septotomy**
0.160	2 (6.3)	32 (2.8)	*Yes*
	26 (2.3)	1108 (97.2)	*No*
			**Precut**
0.404	0 (0)	27 (2.4)	*Yes*
	28 (2.5)	1113 (97.6)	*No*
			**EUS+ERCP**
0.120	5 (4.7)	107 (9.4)	*Yes*
	23 (2.2)	1032 (90.6)	*No*
			**pEP**
0.008	3 (9.7)	31 (2.7)	*Yes*
	25 (2.3)	1109 (97.3)	*No*

Abbreviations: N, number; pEP, post-ERCP aspiration pneumonia; EUS+ERCP, execution of endoscopic ultrasound (EUS) and Endoscopic retrograde cholangiopancreatography (ERCP) during the same anesthesiologist-administered sedation session.

Table S2. (Supplementary Material): Unadjusted analysis on the length of hospital stay expressed in days.

p-value	Hospital LOS mean (SD)	Patients nr. (%)	
/	9.5 (11.3)	1140 (100)	**Included Patients**
			**Sex**
0.010	10.8 (13.9)	487 (42.7)	*Females*
	9.0 (10.6)	653 (57.3)	*Males*
			**Age group nr.**
	9.6 (13.5)	279 (24.5)	*<60 years*
	9.7 (12.7)	316 (27.7)	*60-69 years*
0.126	10.1 (11.1)	334 (29.3)	*70-79 years*
	11.0 (13.5)	211 (18.5)	*>=80 years*
			**Indication to ERCP**
	11.5 (11.3)	572 (50.2)	*Malignant stricture*
	8.8 (14.9)	270 (23.7)	*Biliary Stones*
<0.001	6.8 (7.3)	170 (14.9)	*Benign stricture*
	10.9 (17.2)	128 (11.2)	*Other*
			**Comorbidity**
0.014	10.9 (14.0)	589 (51.7)	Yes
	9.1 (10.8)	551 (48.3)	No
			Diabetes
0.252	10.9 (10.4)	220 (19.6)	*Yes*
	9.8 (13.1)	920 (80.4)	*No*
			Cardiovascular
0.145	10.9 (15.0)	299 (26.2)	*Yes*
	9.7 (11.6)	840 (73.8)	*No*
			Pulmunary
0.387	10.1 (12.8)	95 (8.3)	*Yes*
	9.1 (9.8)	1044 (91.7)	*No*
			**ASA-score**
	8.9 (11.4)	318 (27.9)	1
<0.001	9.1 (10.2)	549 (48.2)	2
	13.0 (16.6)	252 (22.1)	3

	16.2 (21.9)	20 (1.8)	4
			**Cholangitis**
0.025	13.4 (14.2)	87 (7.6)	*Yes*
	9.8 (12.4)	1053 (92.4)	*No*
			**Previous sphyncterotomy**
0.004	8.8 (9.5)	477 (41.9)	*Yes*
	10.9 (14.4)	661 (58.1)	*No*
			**Cannulation**
<0.001	9.5 (11.8)	1053 (92.4)	*Yes*
	16.6 (18.9)	87 (7.6)	*No*
			**Difficult cannulation**
<0.001	13.5 (17.8)	278 (24.4)	*Yes*
	8.8 (10.0)	861 (75.6)	*No*
			**Double wire**
0.251	11.6 (15.5)	92 (8.1)	*Yes*
	9.9 (12.3)	1048 (91.9)	*No*
			**Septotomy**
0.086	14.5 (24.0)	32 (2.8)	*Yes*
	9.9 (12.1)	1108 (97.2)	*No*
			**Precut**
0.480	8.5 (6.1)	27 (2.4)	*Yes*
	10.1 (12.7)	1113 (97.6)	*No*
			**EUS+ERCP**
0.726	10.0 (13.0)	107 (9.4)	*Yes*
	10.4 (8.5)	1032 (90.6)	*No*
			**pEP**
<0.001	26.5 (21.5)	31 (2.7)	*Yes*
	9.6 (12.0)	1109 (97.3)	*No*

Abbreviations: N, number; SD, standard deviation; pEP, post-ERCP aspiration pneumonia; EUS+ERCP, execution of endoscopic ultrasound (EUS) and Endoscopic retrograde cholangiopancreatography (ERCP) during the same anesthesiologist-administered sedation session.
